# Differences in NPI strategies against COVID-19

**DOI:** 10.1007/s11149-022-09452-9

**Published:** 2022-08-22

**Authors:** Margarete Redlin

**Affiliations:** Department of Economics, Paderborn University, Warburger Str. 100, 33098 Paderborn, Germany

**Keywords:** Pandemics, COVID-19, Non-pharmaceutical interventions, Lockdown, Economics, I18, C23

## Abstract

Non-pharmaceutical interventions are an effective strategy to prevent and control COVID-19 transmission in the community. However, the timing and stringency to which these measures have been implemented varied between countries and regions. The differences in stringency can only to a limited extent be explained by the number of infections and the prevailing vaccination strategies. Our study aims to shed more light on the lockdown strategies and to identify the determinants underlying the differences between countries on regional, economic, institutional, and political level. Based on daily panel data for 173 countries and the period from January 2020 to October 2021 we find significant regional differences in lockdown strategies. Further, more prosperous countries implemented milder restrictions but responded more quickly, while poorer countries introduced more stringent measures but had a longer response time. Finally, democratic regimes and stronger manifested institutions alleviated and slowed down the introduction of lockdown measures.

## Introduction

With the outbreak of the COVID-19 pandemic, many countries began implementing contact restrictions to reduce contacts and thus counteract the spread of the virus. Non-pharmaceutical interventions (NPIs) have been and continue to be used as an important tool against Corona. However, the lockdown strategies pursued are not homogeneous across countries. While some countries attempted to counteract the virus with very strict lockdown strategies and measures such as travel bans, school closures, and curfew restrictions even at quite low incidences, other countries largely refrained from imposing mandatory restrictions and merely issued recommendations for action.

Countries in Southeast Asia as well as Australia tried to pursue a zero covid strategy with early border closures, entry barriers, and isolation by imposing a strict lockdown in entire regions even at low incidence levels and trying to eliminate the virus through extensive testing and tracking. Western European countries also show relatively high restrictions. For example, countries such as Germany, France, Italy, and Greece have made vaccination compulsory in certain professions or age levels, and Austria has made it compulsory for the entire adult population. In France, for example, participation in public life is only possible with a health passport, in Austria introduced a lockdown for the unvaccinated, and Germany has been in lockdown several times and the 2G or 2G plus rule applies to participation in public life (2G = only vaccinated or recovered, 2G plus with additional test). Sweden, on the other hand-unlike most of its European neighbors-relied more on voluntarism. And so there were, and still are, primarily only recommendations on how to behave, rather than regulations whose disregard would entail consequences or penalties. And the U.S. version was accompanied by regionally different and in part very strict restrictions. However, these were relaxed early on so as not to harm the economy in the long-term. Thus, it is evident that lockdown strategies across countries were not defined by infection incidence alone. Regional, economic, and institutional factors also appear to be important and thus are the focus of this study.

Our empirical investigation examines the determinants that played a role in setting the lockdown course and analyze country characteristics associated with strict and less strict lockdown strategies. Based on daily panel data for 173 countries and the period from January 1, 2020 to October 23, 2021, we identify the factors that were driving the stringency of the lockdowns. Using GMM and IV techniques to account for a potential endogeneity between the stringency level of NPIs and the spread of the virus and taking into account the actual development of infection and the respective vaccination coverage, our results show that less developed countries and countries with less established institutions and autocratic regimes have adopted harsher lockdown measures. We also identify significant regional differences in the adoption of NPIs. All in all, our findings offer a fruitful contribution to the debate on determinants of NPIs.

The remainder of this paper is organized as follows. Section 2 provides an overview of recent studies and forms the hypotheses for the empirical examination. Section 3 presents the empirical model, the data and our result, and Sect. 4 concludes.

## Literature

The COVID-19 pandemic has resulted in extraordinary burdens for all countries worldwide. To slow the spread of infection, many countries have implemented nonpharmacological interventions. These lockdown measures were primarily aimed at containing the spread of the virus by reducing contacts in the population. Containment was intended to keep the virus and mortality in check and to protect the respective care and health systems from being overburdened.

In this regard, empirical studies provide evidence that lockdown with decreasing mobility in the population is an effective tool for pandemic control. The relationship between decreasing mobility in the population and the incidence of infection during the pandemic has been clearly demonstrated empirically. A reduction in mobility has been shown to lower the reproductive numbers (Nouvellet et al., 2021). There is also empirical evidence conforming that the overall set of nonpharmacological interventions had the desired effect on the incidence of infection and thus on the mortality rates (Hsiang et al., 2020). Cross country studies show that lockdown is effective in reducing the number of new cases in the countries that implement it compared with those countries that do not (Bo et al., 2021, Alfano & Ercolano, 2020, Banholzer et al., 2020, Hartl 2020). Flaxman et al. (2020) model how many infections and deaths were prevented by the non-pharmacological interventions and lockdowns in 11 European countries by May 2020, with the result that more than 3 million lives were saved. And Askitas et al. (2021) analyses worldwide effects of non-pharmaceutical interventions on COVID-19 incidence and population mobility patterns using a multiple-event study confirming that lockdown had significant effects on reducing COVID-19 infections.

However, a lockdown has not only desirable effects, but also negative effects and high psychological, social, and economic costs (Bonaccorsi et al., 2020). Thus, the negative side effects and benefits must be weighed when introducing it (Layard et al., 2020). Thus, some countries implemented strict measures only intermittently and only when viral incidence was high, and strategies were not always consistent when infection histories were similar.

**Fig. 1:**
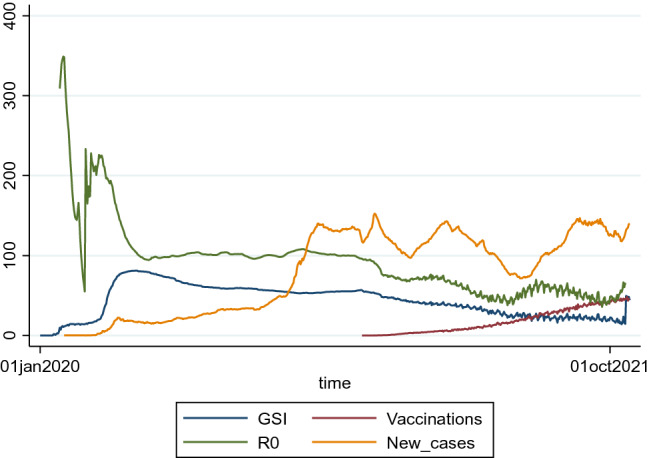
Worldwide averages in new COVID-19 cases (per million), COVID-19 reproduction rate (*100) R0, GSI and vaccination rate (%)

In general, we would expect the extent of contact restrictions to be higher the more severely the country is affected by the pandemic event, and the measures to be relaxed as incidences decline. This would be reflected in a positive correlation between lockdown and infection rates. Researchers at Oxford University developed the Government Stringency Index (GSI) during the COVID-19 pandemic, which quantifies the severity of lockdowns in states worldwide. It captures all Corona restrictions in place, such as school closures, closed workplaces, travel restrictions, or contact restrictions in a country, and combines them into one index. The correlation coefficient based on data from 173 countries from January 2020 to October 2021 between the GSI and reported new COVID-19 cases is 0.1465. The correlation is relatively low, indicating that only a small proportion of the variance in the Stringency Index can be explained by the prevailing incidence of infection. Figure 1 shows the development of the global averages in daily reported COVID-19 cases, the COVID-19 reproduction rate R0, the Government Stringency Index and the vaccination rate in the population. While the global outbreak at the beginning of 2020 led to a dramatic increase in stringency, no joint movement of COVID-19 cases and the Government Stringency Index is visible in the wider context. The development of the reproduction rate also shows no clear correlation with the policy measures. Thus, lockdown strategies cannot be explained by infection rates alone, but other factors also seem to play an important role in governments' decisions.

The determinants of lockdown policies have been considered only sparsely in the literature (Aksoy et al., 2020; De Simone & Mourao, 2021; Ferraresi et al., 2020; Frey et al., 2020). De Simone and Mourao (2021) analyze the relationship between country characteristics and lockdown timing and find that urban population and political stability are conducive to a prompt activation of a government’s lockdown policy after initial cases while a country’s wealth and the rule of law may produce an opposite effect and be an obstacle to an immediate policy activation. Aksoy et al. (2020) show that countries with high levels of public attention to COVID-19 are more likely to implement non-pharmaceutical interventions. Analyzing political determinants of lockdown differences in 110 Frey et al. (2020) find that autocratic regimes imposed more stringent lockdowns. They show that in authoritarian countries, a doubling of cases is associated with an increase in stringency 17% higher than in democracies. Ferraresi et al. (2020) provide an event-study design which analyses the determinants of differences in timing and intensity of stringency measures undertaken. By analyzing dummies for economics, political and institutional characteristics, they show the different trajectories of lockdown measures depending on the dummies and find that, for the same number of cases identified countries characterized by low political stability low level of development, low level of digitalization and a high degree of decentralization have adopted less stringent measures. Further, openness and being in an electoral year is associated with a more stringent lockdown policy.

Our paper extends these analyses by offering a precise and differentiated review of the factors associated with the implementation of NPIs for reducing COVID-19. To examine the determinants differences in lockdown strategies, a holistic view across countries and over time is needed, including not only infection incidence but also regional and country-specific aspect in terms of development status as well as institutional characteristics. In addition, the inclusion of vaccination coverage is relevant, as it is expected that with higher vaccination coverage, lockdown measures can be relaxed. This study, therefore, uses panel regression analysis with daily observations of the Government Stringency Index, reported new COVID-19 cases, proportion of the population vaccinated and regional, economic, political, and institutional determinants to analyze which factors have an impact on national lockdown strategies.

## Empirical evidence

### Estimating model

In general, implementation of NPIs can be expected to be related to COVID-19 development. If incidence increases, it can be expected that the government will, on average, introduce harsher restrictions to limit contacts in the population. This effect may be mitigated if a large portion of the population is already vaccinated. Vaccinated individuals generally have high protection against the disease and tend to show mild disease courses even when vaccine breakthroughs occur. Following this reasoning our starting point is a model of the form

$${GSI}_{i,t}=\alpha +{\beta }_{1} {cases}_{i, t}+ {\beta }_{2} {vaccinations}_{i,t-1}+{\beta }_{3} {x}_{i,t}+ {\mu }_{i}+{\epsilon }_{i,t}$$

where ${GSI}_{i,t}$ represents the composite Government Stringency Index in country i at day t, ${cases}_{i, t}$ is the number of new COVID-19 cases per million people in country i at day t, and ${vaccinations}_{i,t}$, is the percent of people fully vaccinated against COVID-19 in country i at day t, and the disturbance term is composed of the individual effect ${\mu }_{i}$ and the stochastic disturbance ${\epsilon }_{i,t}$. We account for regional, economic, political, and institutional differences characteristics by including additional specific explanatory variables that capture these characteristics in ${x}_{i,t}$.

### Data

Our analysis is based on unbalanced panel dataset of daily data covering 173 countries from the period January 1, 2020 to October 23, 2021.

### Dependent variable

Our dependent variable *GSI* is the COVID-19 Government Stringency Index. It is a composite measure calculated by The Oxford Coronavirus Government Response Tracker (OxCGRT) project based on nine government response indicators.^1^ The nine metrics used to calculate the Stringency Index are: school closures; workplace closures; cancellation of public events; restrictions on public gatherings; closures of public transport; stay-at-home requirements; public information campaigns; restrictions on internal movements; and international travel controls. The index on any given day is calculated as the mean score of the nine metrics, each taking a value between 0 and 100. A higher score indicates a stricter response (i.e., 100 = strictest response).

### Baseline explanatory variables

First, we include the 7-day rolling average of new daily confirmed *cases per million* people. The data comes from the COVID-19 Data Repository by the Center for Systems Science and Engineering (CSSE) at Johns Hopkins University (JHU).^2^

Additionally, to the level of the infection, we control for the dynamics of COVID-19 by including estimates of the *reproduction rate R0* (Arroyo-Marioli et al., 2021). The reproduction rate represents the average number of new infections caused by a single infected individual. Thus, if the rate is greater than 1, the infection is able to spread, while the number of cases will gradually decrease, if the rate is below 1.

Further we account for the effect of *vaccinations*. This variable is defined as the total number of people who received all doses prescribed by the vaccination protocol per 100 people in the total population. The data is provided by the COVID-19 Our World in Data project and is based on public official sources.

### Regional characteristics

We control for regional differences by including dummies for continents in the regression on the one hand and running the regressions separately for the individual continents on the other. The dummies represent *Africa*, *Asia*, *Europe*, *North America*, *Oceania*, and *South America*.

### Development

First, we investigate the effect of development on the stringency by including $GDP per capita$. We use gross domestic product at purchasing power parity (constant 2011 international dollars) from the World Bank World Development Indicators.

Second, we include *extreme poverty* measured as the share of the population living in extreme poverty from the World Bank World Development Indicators.^3^

Finally, we use the *Human Development Index* provided by the United Nations Development Programme (UNDP) to account for the effect of development. The HDI is a composite index measuring average achievement in three basic dimensions of human development-a long and healthy life, knowledge, and a decent standard of living.

### Institutional and political characteristics

We use the Worldbank’s Worldwide Governance Indicators which measure six broad dimensions of governance to investigate the effect of institutions.^4^ The six dimensions include:

*Voice and Accountability,* which captures perceptions of the extent to which a country's citizens are able to participate in selecting their government, as well as freedom of expression, freedom of association, and a free media.

*Political Stability and Absence of Violence/Terrorism*, a measure of perceptions of the likelihood of political instability and/or politically motivated violence, including terrorism.

*Government Effectiveness*, which captures perceptions of the quality of public services, the quality of the civil service and the degree of its independence from political pressures, the quality of policy formulation and implementation, and the credibility of the government's commitment to such policies.

*Regulatory Quality*, a measure of perceptions of the ability of the government to formulate and implement sound policies and regulations that permit and promote private sector development.

*Rule of Law*, accounting for the extent to which agents have confidence in and abide by the rules of society, and in particular the quality of contract enforcement, property rights, the police, and the courts, as well as the likelihood of crime and violence.

And *Control of Corruption*, which captures perceptions of the extent to which public power is exercised for private gain, including both petty and grand forms of corruption, as well as "capture" of the state by elites and private interests. The six indicators are reported in their standard normal units, ranging from approximately -2.5 to 2.5.

Table 1 shows the descriptive statistics of all variable.

**Table 1:** Descriptive statistics

Variable	Obs	Mean	Std. dev	Min	Max
GSI	104,655	56.734	20.635	0	100
New cases (per million)	117,314	84.885	165.706	− 272.971	3385.473
Reproduction rate R0	100,726	1,002	0.344	− 0.030	5.960
Vaccinations (per hundred)	25,445	21.870	22.792	0	118.12
GDP per capita	111,498	19,244.6	20,057.16	661.24	116,935.6
Extreme poverty	74,606	13.499	19.991	0.1	77.6
HDI	111,185	0.726	0.150	0.394	0.957
Voice and accountability	111,738	− 0.041	0.987	− 2.159	1.725
Political stability	112,281	− 0.079	0.977	− 2.731	1.913
Government effectiveness	110,885	− 0.002	1.004	− 2.344	2.335
Regulatory quality	110,885	− 0.003	0.993	− 2.340	2.206
Rule of law	110,885	− 0.025	0.995	− 2.346	2.079
Control of corruption	110,885	− 0.012	1.013	− 1.905	2.270

### Regression results

Table 2 presents the results of our baseline specification. The results from ordinary least squares (OLS) estimation and fixed effect (FE) estimation are provided in column (1) and (2). Both coefficients show the expected results. The coefficient for new cases is positive and highly significant. It is thus evident that, on average, the increase in COVID-19 cases is associated with the introduction of harsher NPIs to reduce the contacts and counteract the spread of the virus. The coefficient for the vaccination rate, on the other hand, is negatively significant. This indicates that the lockdown measures could be relaxed as the vaccination rate increased–holding all other factors constant. This result is consistent with Patel et al. (2021) modeling that NPIs and vaccination coverage are both levers that can be used to control spread and showing that with increasing vaccination rate, the restrictions on NPIs can be relaxed.

**Table 2:** Baseline regression

	(1)	(2)	(3)	(4)	(5)
	OLS	FE	GMM	Lewbel IV	Panel event
New cases (per million)	0.0150^***^	0.0139^***^	0.0132^***^	0.0143^***^	0.0117^***^
	(0.003)	(0.003)	(0.003)	(0.003)	(0.003)
Vaccinations (per hundred)	− 0.2306^***^	− 0.3301^***^	− 0.3624^***^	− 0.3326^***^	− 0.2682^***^
	(0.034)	(0.035)	(0.051)	(0.036)	(0.054)
*R2*	0.1396	0.3485	–	0.3485	0.3751
*Hansen j*	–	–	144.11 0.889	3.66 0.160	–
*AR2*	–	–	−1.22 0.223	–	–
*Instruments*	–	–	169	2	–
*Countries*	173	173	173	173	173
*Obs*	20,258	20,258	20,258	20,189	20,258

Estimates based on (1) OLS, (2) fixed effects, (3) two-step system GMM, (4) Lewbel instrumental variables regressions and Clarke and Tapia-Schythe (2021) panel event study estimation. R2 denotes the coefficient of determination. Hansen j denotes the Hansen test statistic for overidentifying restrictions. AR2 denotes the Arellano and Bond second order serial correlation test. Dependent variable is the Government Stringency Index. Clustered standard errors in parentheses; for GMM Windmeijer (2005) standard errors

*p < 0.10, **p < 0.05, ***p < 0.01

In a second step we provide robustness checks regarding potential endogeneity and the event character of the data. Technically, endogeneity occurs when explanatory variables in a regression model are correlated with the error term. This can occur (1) when important variables are omitted from the model and (2) in case of reverse causality. In our model, the Stringency Index is not only an outcome of the corona cases but can also help to explain the further course of the cases as a predictor. The policy measures serve to reduce the contacts, which in turn have an impact on the spread of the virus. The issue of endogeneity can be addressed by using instrumental variables. This potential endogeneity could bias our OLS and FE results. Therefore, we use the system generalized method of moment (GMM) estimator developed by Blundell and Bond (1998), which relies on a set of “internal” instruments contained within the panel itself. Further we use the instrumental variable (IV) approach developed by Lewbel (2012). The estimator exploits model heteroscedasticity to construct internal instruments using the available regressors. In addition, external instruments can be added to improve the efficiency. Finally, we apply a panel event study based on the approach of Clarke and Tapia-Schythe (2021). We define the event as the COVID-19 outbreak in a given country to control for the fact that the virus did not break out at the same time in the individual countries. The virus outbreak is defined as the time when more than ten people in the country were infected for the first time. The three estimators confirm our previous OLS and FE findings.

When looking at the coefficient of determination, it is apparent that only about one third of the variance in the NPIs can be explained by differences in infection and vaccination rates. Thus, there seem to be other important factors responsible for the lockdown policy and implementation. In the following, we will take a closer look at regional, economic and institutional factors.^5^

**Fig. 2:**
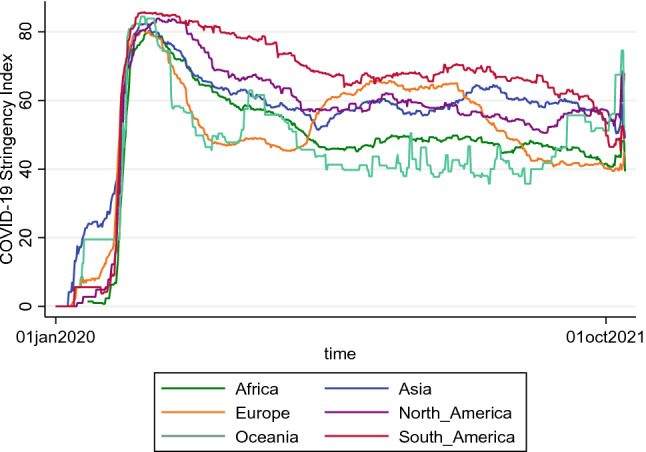
Regional differences in the GSI

Figure 2 shows the development of the GSI for the individual regions. It is evident that strict measures were introduced worldwide with the outbreak of the virus in order to keep the spread within limits. However, the further courses show regional differences. While the GSI is at a relatively high level in South and North America and Asia, Africa and Oceania show on average lower values. In Europe, there are greater fluctuations in the GSI.

While the illustration shows regional differences without taking other factors into account, we will further examine the differences taking into account the prevailing incidences and vaccination rates. We take a closer look at regional differences in the introduction of NPIs by (a) including regional dummies, (b) running the baseline regression separately for individual regions, and (c) presenting regional differences for the margins for different vaccination rates and incidences. The regression results are presented in Table 3.

**Table 3:** Regional differences in the government stringency index

	(1)	(2)	(3)	(4)	(5)	(6)	(7)
		Africa	Asia	Europe	North America	Oceania	South America
New cases (per million)	0.0080^***^	0.0164^***^	0.0151^***^	0.0129^***^	0.0149^***^	− 0.0088	− 0.0000
	(0.000)	(0.002)	(0.001)	(0.000)	(0.001)	(0.010)	(0.001)
Vaccinations (per hundred)	− 0.3777^***^	− 0.3924^***^	− 0.2191^***^	− 0.4335^***^	− 0.1160^***^	0.8732	− 0.3477^***^
	(0.002)	(0.029)	(0.009)	(0.004)	(0.009)	(0.054)	(0.013)
Africa	5.7658^***^	–	–	–	–	–	–
	(0.275)	–	–	–	–	–	–
Asia	7.952^***^	–	–	–	–	–	–
	(0.347)	–	–	–	–	–	–
Europe	− 5.065^***^	–	–	–	–	–	–
	(0.291)	–	–	–	–	–	–
North America	6.145^***^	–	–	–	–	–	–
	(0.346)	–	–	–	–	–	–
Oceania	44.071^***^	–	–	–	–	–	–
	(0.752)	–	–	–	–	–	–
Hansen j	142.68	34.38	37.29	41.71	− 0.70	0.63	6.49
	0.847	0.875	0.678	0.396	0.481	0.960	0.690
AR2	− 1.37	0.70	− 1.33	− 0.75	14.24	1.00	− 1.04
	0.171	0.486	0.184	0.452	0.507	0.316	0.299
Instruments	169	48	45	43	18	7	12
Countries	173	48	45	43	18	7	12
Obs	20,258	1794	5212	8482	2193	435	2142

Estimates based on two-step system GMM regressions. Hansen j denotes the Hansen test statistic for overidentifying restrictions. AR2 denotes the Arellano and Bond second order serial correlation test. Dependent variable is the Government Stringency Index. Robust Windmeijer (2005) standard errors in parentheses

*p < 0.10, **p < 0.05, ***p < 0.01

It should be taken into account that the observations are lower for less developed countries due to the limited availability for the vaccination variable. While the observations in Europe and North America are largely available, there are large gaps for Africa, Asia and Oceania. This selection bias can lead to coefficient bias and low significance and should be taken into account in the further analysis. While the figure shows regional differences, the regressions additionally control for incidence and vaccination coverage. The coefficients of regional dummies show that regional characteristics have significant effects on the introduction of NPIs.

Controlling for the number of cases and for vaccination coverage, Oceania shows the harshest measures. The results mirror the stringent policy in countries such as Australia, New Zealand, and many small Pacific Island nations where governments placed the countries in a nationwide lockdown and closed their international borders with already low case numbers. Asian countries also show comparatively high restrictions. Compared to South America, which is taken as the reference region, the lockdown indicator for Asian countries is on average eight points higher (on a scale from 0 to 100). This reflects the fact that Oceania and Asia have been pursuing zero-covid strategies for a long time and have relied on very stringent measures to prevent the spread. European countries, however, show on average less harsh lockdown measures, when controlling for cases and vaccination rates. The score is five points below the control region.

Looking at the development as a function of case numbers and the introduction of vaccination, the following specifications show that the responses to increasing case numbers and to increasing vaccination coverage were different in different regions. African countries show the strongest response with respect to lockdown measures when case numbers increase, followed by Asian and North American countries. In Europe, lockdowns appear to be influenced by case numbers to a smaller extent, and in South America and Oceania, lockdowns appear to respond more to global events than to the situation in the country itself, as case numbers do not show significant effects here. Vaccination rates are associated with the largest effects on NPIs in Europe followed by Africa and South America, implying that this is where the highest relaxations of lockdowns measures were seen as vaccination rates increased. Asia and North America show substantially lower effects here. Regional differences in the implementation of NPIs may also reflect differences in population density, rural-urban dimensions, and population age structure. As shown by Kashnitsky and Aburto (2020) differences in the population structure have significant effects to the magnitude of the pandemic, thus, they could also have effects on policy measures.

**Fig. 3:**
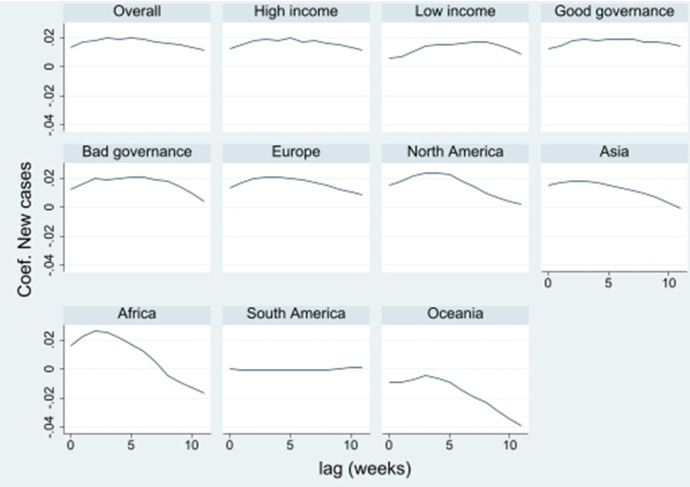
Effect of new COVID-19 cases lagged by 1 to 12 weeks

**Fig. 4:**
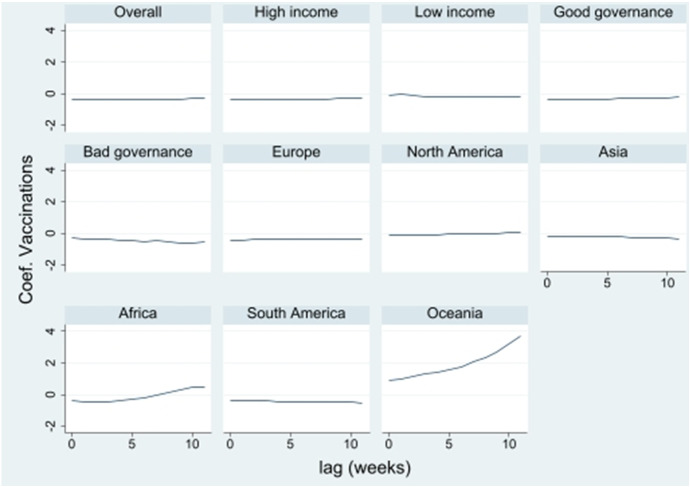
Effect of the vaccination rate lagged by 1 to 12 weeks

To examine the reaction time from stimulus to response, we analyze the effects of the time-lagged explanatory variables. Figures 3 and 4 show how the effects behave for a lag of 1–12 weeks. In general, we see that the effect for new COVID-19 cases first increases and then decreases with time. For the entire panel, the peak is observed after three weeks. This means that it takes some time to implement the measures. Looking at the subpanels, it is evident that the response time is faster in richer countries and regional differences are also apparent.^6^ From a lag of 10 weeks, the significance of the effects drops sharply, which means that the case numbers from 10 weeks ago and before no longer show significant effects on today's measures.

For vaccinations, the effect behaves relatively constant.^7^ This may be due to the fact that the development of the vaccination rate shows no jumps and little variation within these short periods.

In the next step, additionally to just account for case numbers and vaccination rate, we include different controls for COVID-19 development, economic development and institutions to account for these factors. First, additionally to the level of the infection, we control for the dynamics of COVID-19 by including estimates of the reproduction rate R0 (Arroyo-Marioli et al., 2021). The results with the reproduction rate in exchange for the number of cases and supplementary to the number of cases are presented in specifications (1) and (2) in Table 4.

**Table 4:** Institutional and Political Effects in the Government Stringency Index

	(1)	(2)	(3)	(4)	(5)	(6)	(7)	(8)	(9)
New cases (per million)	0.0139^***^	–	0.0129^***^	0.0003^***^	0.0061^***^	0.0004^***^	0.0133^***^	0.0131^***^	0.0128^***^
	(0.005)	–	(0.002)	(0.000)	(0.000)	(0.000)	(0.000)	(0.000)	(0.000)
Vaccinations (per hundred)	− 0.3386^***^	− 0.3649^***^	− 0.3635^***^	− 0.5082^***^	− 0.4467^***^	− 0.5001^***^	− 0.3575^***^	− 0.3582^***^	− 0.3581^***^
	(0.043)	(0.052)	(0.000)	(0.002)	(0.002)	(0.002)	(0.002)	(0.002)	(0.002)
Reproduction rate R0	9.7230	10.6117	–	–	–	–	–	–	–
	(7.000)	(7.023)	–	–	–	–	–	–	–
High income country	–	–	− 2.8428^***^	–	–	–	–	− 1.8100^***^	0.5167
	–	–	(0.134)	–	–	–	–	(0.164)	(0.346)
ln GDP pc	–	–	–	− 16.3301^***^	–	–	–	–	–
	–	–	–	(0.170)	–	–	–	–	–
Extreme poverty	–	–	–	–	0.5506^***^	–	–	–	–
	–	–	–	–	(0.035)	–	–	–	–
HDI	–	–	–	–	–	− 149.5101^***^	–	–	–
	–	–	–	–	–	(2.305)	–	–	–
Good governance	–	–	–	–	–	–	− 3.5108^***^	− 2.5101^***^	− 0.6822^**^
	–	–	–	–	–	–	(0.182)	(0.132)	(0.285)
High income * Good governance	–	–	–	–	–	–	–	–	− 3.4041^***^
	–	–	–	–	–	–	–	–	(0.237)
Hansen j	159.31	163.21	146.25	140.35	99.87	138.21	− 1.22	143.61	142.20
	0.523	0.459	0.791	0.878	0.787	0.921	0.222	0.819	0.826
AR2	− 0.09	− 0.07	− 1.23	− 1.17	− 0.72	− 1.18	144.83	− 1.23	− 1.24
	0.931	0.944	0.218	0.241	0.470	0.236	0.815	0.217	0.213
Instruments	165	165	165	165	116	167	165	165	165
Countries	169	169	173	166	116	168	173	173	173
Obs	19,944	19,944	20,258	19,690	14,311	19,988	20,258	20,258	20,258

Estimates based on two-step system GMM regressions. Hansen j denotes the Hansen test statistic for overidentifying restrictions. AR2 denotes the Arellano and Bond second order serial correlation test. Dependent variable is the Government Stringency Index. Robust Windmeijer (2005) standard errors in parentheses

*p < 0.10, **p < 0.05, 0***p < 0.01

There is no significant correlation between the reproduction rate and the lockdown measure. This may be due to the fact that the reproduction rate measures the development but not the level of the pandemic. Thus, our results suggest that policy measures are responding to the current level of the pandemic rather than the short-term trend.

In the following we examine development related differences. Development is measure by four different indicators-a dummy for high income countries, GDP per capita, the share of people living in extreme poverty, and the HDI. The results are presented in Table 4 in specification (3) to (6) and show that developed countries-measured by GDP per capita and HDI adopted less stringent measures, while the reactions of poorer countries are more stringent. High income countries show an GSI index value that is on average three percentage points higher. Our result is in line with De Simone and Mourao (2021) who show that richer countries tended to take longer to establish a “lockdown”. Our results suggest that economic lockdown costs are much higher in developed countries, thus many industrialized countries may hesitate to interrupt their economic activities and establish lockdowns due to their related economic costs or were eager to ease back the NPIs relatively quickly after a brief lockdown. However, Ferraresi et al. (2020) find contradictory findings and argue that in the initial phase of the pandemic developed countries adopted more stringent measures as compared to developing ones. This result, though, is only significant in the initial phase of the pandemic.

To shed more light on the effects of institutions and state characteristics we estimate the effects of the Worldbank’s Worldwide Governance Indicators on the Government Stringency Index. Specification (7) in Table 4 presents the results where all governance indicators are summarized in one “good governance” dummy. The dummy is one if the country's institutional quality is above average (> 0).^8^ Countries with a well-functioning institutional apparatus had lockdown values that were on average 3.5 points lower. In line with previous findings (De Simone & Mourao, 2021) our results show that high quality of institutions can have a detrimental effect on the implementation of strict measures and on response time. Sophisticated institutional processes can slow down implementation, for example through a bloated legal system and high bureaucratic burdens and thus hinder effective execution. Furthermore, government authorities may need to limit certain human rights in these national emergencies when combating the spread of the virus. This is more difficult the more these rights are enshrined in national jurisprudence and laws. Therefore, democratic regimes may find more obstacles to imposing mandatory and harsh measures while autocratic countries face fewer administrative hurdles and can enforce measures more quickly and with less resistance (De Simone & Mourao, 2021; Frey et al., 2020).

Since it is often the richer countries that are also institutionally better off, we go on to test the joint effect of the two to see whether it is income, the institutional framework, or the combination of the two that makes more of a difference. Therefore, we introduce models in which we estimate the two dummy variables for good governance and high income together and additionally together with their interaction. While the estimates with both variables still show significant negative coefficients, the results change with the inclusion of the interaction. Looking at the interaction, we see that countries that have both high income and are institutionally well-positioned had lower constraints on average. Good governance by itself still shows a negative effect, but the effect size and significance are now smaller. It is evident that when considering income and institutions jointly, income alone does not have a significant effect on policy measures. Thus, it is more the institutional framework, especially in combination with higher income, that matters.

Overall, our results show that differences in lockdown strategies can be explained only to a limited extent by differences in infection numbers and vaccination rates. Rather, we show that regional differences co-determine lockdown strategies. This is in line with Petherick et al. (2020) who show that the development of the number of cases and the response of the state is not parallel. Rather the stringency of policy response has varied substantially, with many coutries experiencing a rise in cases in the summer and fall even as levels of stringency remained approximately constant or fell. Similar results were also found for the US, where Hallas et al (2021) find significant variation in both the measures that states adopt and when they adopt them. Their results shows that after initial peaks in stringency, policy variation by state, region, and political affiliation continued into the fall, with Northeastern and Democrat-led states experiencing more stringent responses overall.

In order to better compare the results and their quantitative significance, we present the effects in relation to the standard deviations in Table 5. In addition to the coefficients and standard deviations of the explanatory and dependent variable, the standardized coefficients are presented. The coefficients for COVID-19 cases shows that the change by one standard deviation increases the Government Stringecy Index by 22 units, i.e., about 17% of its standard deviation. In contrast, a one standard deviation change in the vaccination rate has an effect that is three times as large, at 50%. The effects on income and government quality are relatively small. However, the combination of both leads to a 10% change in the standard deviation of stringency.

**Table 5:** Standardized estimates

	(1)	(2)	(3)	(4)	(6)	(7)
	Coef	Std dev *X*	Std dev *Y*	*x*-stand. coefficient	*y*-stand. coefficient	Fully stand. coefficient
New cases (per million)	0.0128^***^	205.009	15.7197	22.0180	1,228.1016	0.1669
	(0.000)	–	–	–	–	–
Vaccinations (per hundred)	-0.3581^***^	22.1125	15.7197	− 7.9185	− 43.8975	− 0.5038
	(0.002)	–	–	–	–	–
High income country	0.5167	0.3630	15.7197	0.1876	30.4233	0.0119
	(0.346)	–	–	–	–	–
Good governance	− 0.6822^**^	0.1979	15.7197	− 0.1350	− 23.0427	− 0.0086
	(0.285)	–	–	–	–	–
High income * Good governance	-3.4041^***^	0.4959	15.7197	− 1.6881	− 4.6179	− 0.1074
	(0.237)	–	–	–	–	–
Obs	20,258	–	–	–	–	–

*p < 0.10, **p < 0.05, ***p < 0.01

In summary, we show that economic, institutional, and political factors of the country play a significant role in the implementation and harshness of interventions. In the future, governments at all levels would benefit from adopting an evidence-based approach to the actions they take.

## Concluding remarks

NPIs are an effective strategy in combating the COVID-19 pandemic. Recent studies show that a suitable combination of NPIs is necessary to curb the spread of the virus (Haug et al., 2020) and that vaccination alone is insufficient to contain the outbreak (Moore et al., 2021). However, the timing and stringency to which these measures have been implemented varied between the countries and regions. The differences in stringency can only be explained to a limited extent by the number of infections and the prevailing vaccination strategies. Our study aims to shed more light on the lockdown strategies and to identify the determinants underlying the differences between countries on regional, economic, institutional, and political level. Based on daily panel data for 173 countries and the period from January 2020 to October 2021, we analyze the factors that were driving the stringency of the lockdowns. We identify significant regional differences. It is evident that some regions and countries were more responsive to global developments of the pandemic, while others adjusted their NPI measures more on country specific virological development. Asian countries introduced relatively strict measures, which were not directly related to domestic infection rates. In North America and Europe, on the other hand, the stringency of the lockdown was comparatively small. An investigation of the relationship between economic development and NPIs shows that the associated high economic lockdown costs in high developed countries led to a weakened lockdown reaction, while poorer countries-in terms of GDP per capita, poverty level and HDI-have introduced more stringent measures. On the other hand, wealthier countries showed a quicker response, while in poorer countries the response time was longer. Further, democratic regimes and stronger manifested institutions alleviated and slowed down the introduction of lockdown measures. In most political systems and administrative organizations, there was maximum uncertainty about pandemic response and the introduction of NPIs. Our results indicate, that for the future a more structured pandemic policy is needed that provides quick and clear guidelines and recommendations for action. Better conceptual, personnel and material recourses are important prerequisites for fast and effective pandemic response. Rapid and consistent implementation and targeted adaptation of NPIs can both save lives and reduce lockdown duration. Preventive measures, such as the installation of air filters in workplaces and schools and comprehensive testing strategies, may also reduce the need for NPIs and the associated economic and social costs.

Overall, our analysis makes a valuable contribution to the discussion of lockdown determinants. However, our estimation is limited by the type of data utilized. Although we control for temporal effects, it should be noted that we do not explicitly control for the prevailing virus variants, since this kind of data is not available in the panel format. It is also important to note, that the study does not take into account the Omicron wave, as only the time period until October 2021 is considered.

## Appendix: 1 Table A1 Mean values for specific groups

**Table:**

Variable	Overall	Africa	Asia	Europe	North America	Oceania	South America	High income (yes)	High income (no)	Good governance (yes)	Good governance (no)
GSI	56.734	52.694	60.028	54.071	60.645	46.900	68.583	58.055	55.325	55.976	57.353
New cases (per million)	84.885	26.580	74.692	162.56	86.939	15.839	120.054	124.203	39.594	122.914	50.193
Vaccinations (per hundred)	21.870	6.533	21.983	26.22	25.363	18.705	19.701	25.448	10.420	26.028	23.770
GDP per capita	19244.6	5488.3	23862.9	33478,5	19231.3	14170.3	13885.9	33206.6	5179.0	31393.9	9046.9
Extreme poverty	13.499	33.836	5.667	0.901	5.599	8.359	2.845	1.268	22.363	5.397	19.143
HDI	0.726	0.562	0.742	0.880	0.757	0.731	0.764	0.840	0.610	0.832	0.642
Voice and accountability	− 0.041	− 0.615	− 0.667	0.828	0.413	0.799	0.230	0.298	− 0.415	0.763	− 0.677
Political stability	− 0.079	− 0.691	− 0.391	0.549	0.455	0.768	− 0.190	0.301	− 0.500	0.730	− 0.726
Government effectiveness	− 0.002	− 0.772	0.073	0.868	0.101	0.152	− 0.272	0.551	− 0.603	0.874	− 0.684
Regulatory quality	− 0.003	− 0.770	0.006	0.940	0.147	0.078	− 0.317	0.517	− 0.566	0.853	− 0.668
Rule of law	− 0.025	− 0.699	− 0.102	0.855	0.076	0.491	− 0.390	0.485	− 0.579	0.874	− 0.725
Control of corruption	− 0.012	− 0.635	− 0.164	0.808	0.127	0.590	− 0.238	0.484	− 0.550	0.907	− 0.727
Polity2	4.291	2.889	0.188	8.750	7.177	3.237	6.969	4.975	3.615	7.825	2.068
Military exp (in GDP)	1.982	1.791	3.070	1.678	1.067	1.464	1.718	2.253	1.710	1.860	2.068

## Footnotes

1 https://www.bsg.ox.ac.uk/research/research-projects/covid-19-government-response-tracker.

2 https://coronavirus.jhu.edu/map.html.

3 https://datatopics.worldbank.org/world-development-indicators/.

4 https://info.worldbank.org/governance/wgi/.

5 In the further course, additional country characteristics are included in the model. Since these variables do not change over the daily observations for the individual countries, they would be dropped from the model by the FE or the Lewbel estimator. For this reason, the following regressions are based on the GMM estimator.

6 It should be noted that the effects for South America and Oceania are not significant.

7 The effect for Oceania is not significant.

8 In addition, we also test for the effects of the individual indices of the governance indicator. All sub-indices confirm our findings and show highly significant negative correlations with the GSI.
